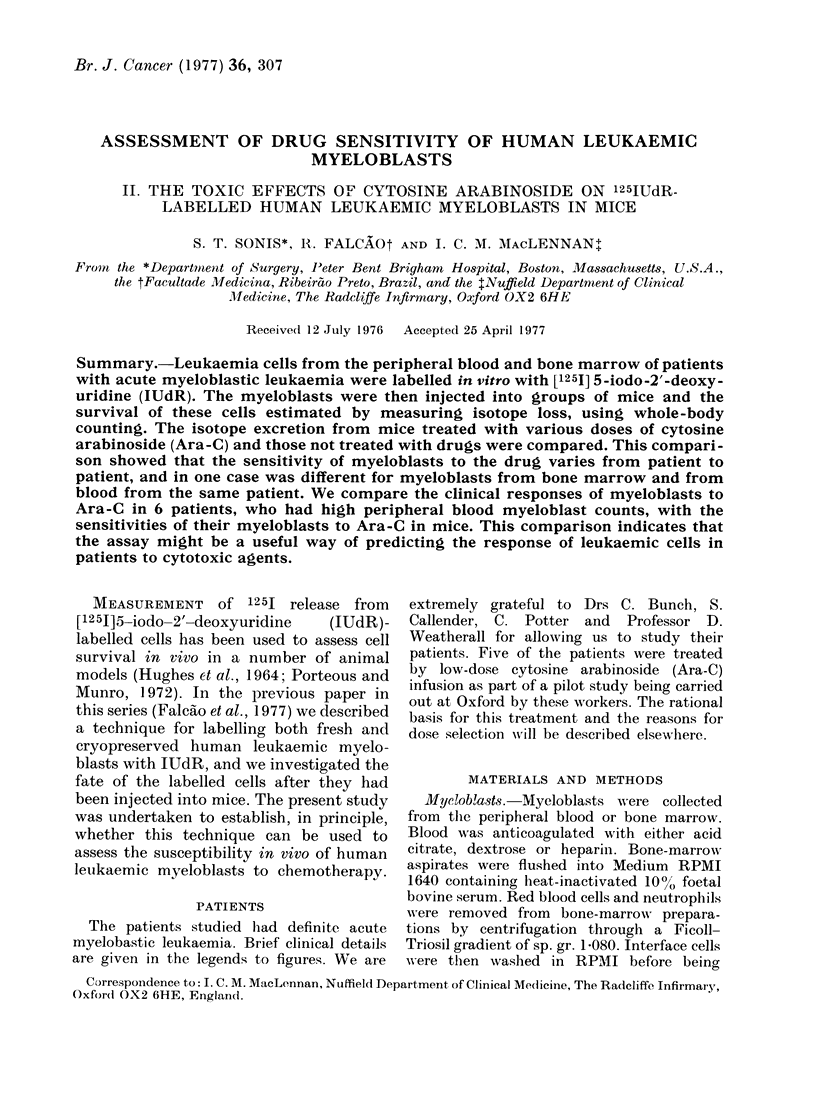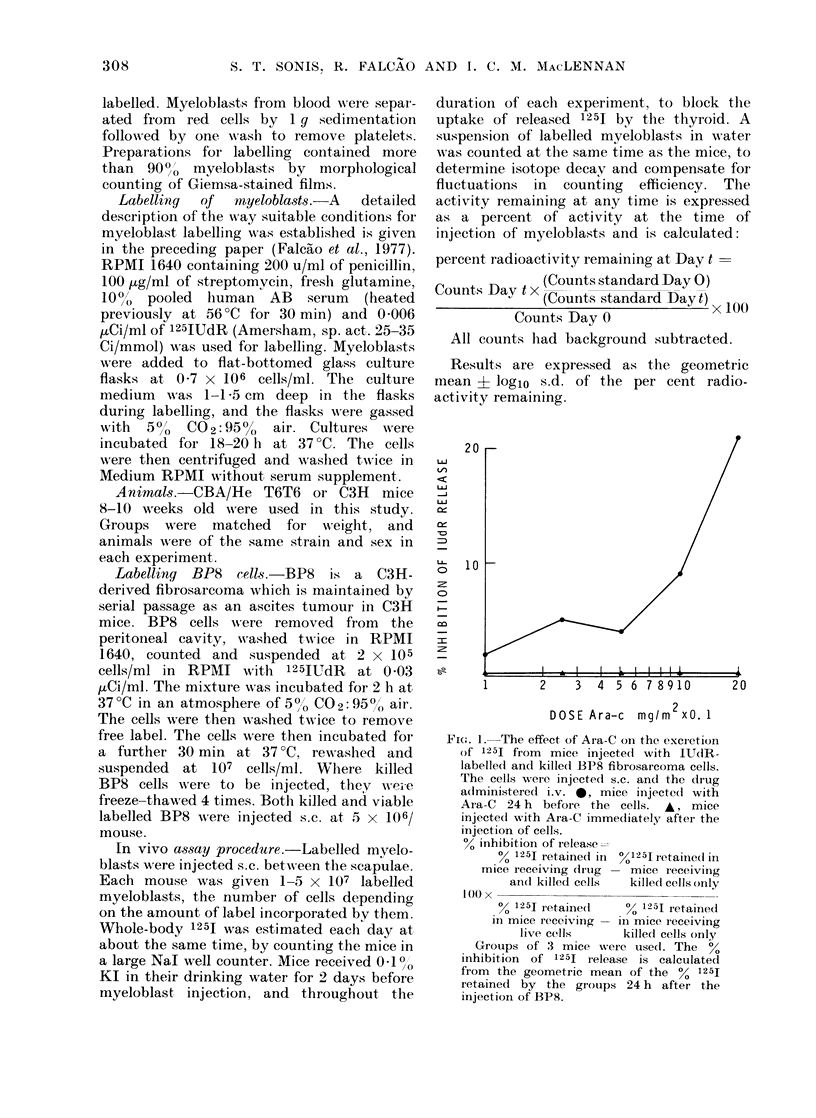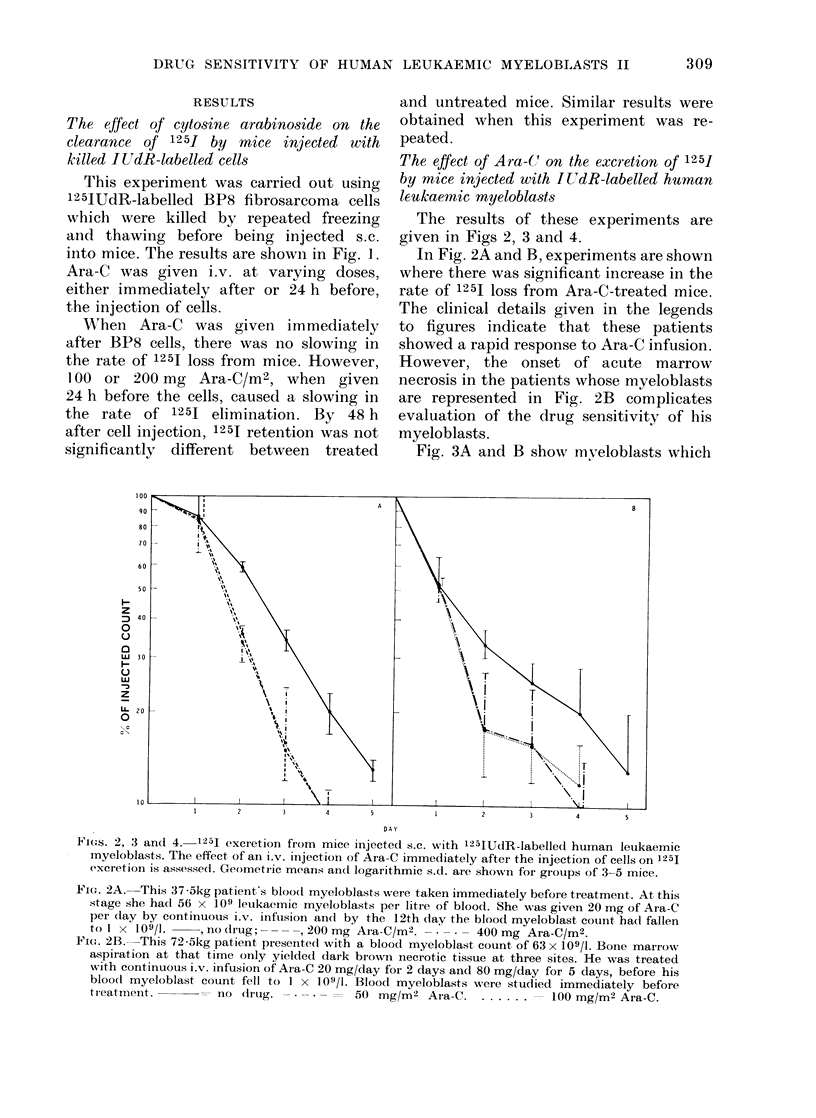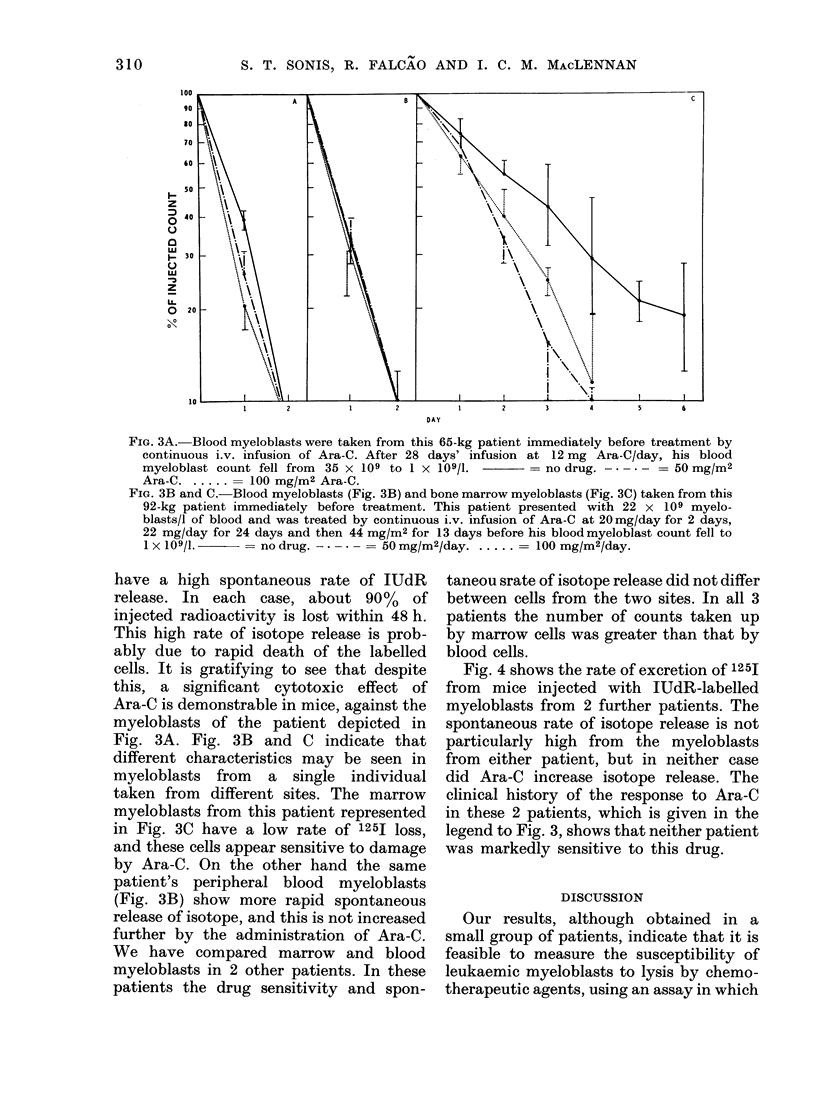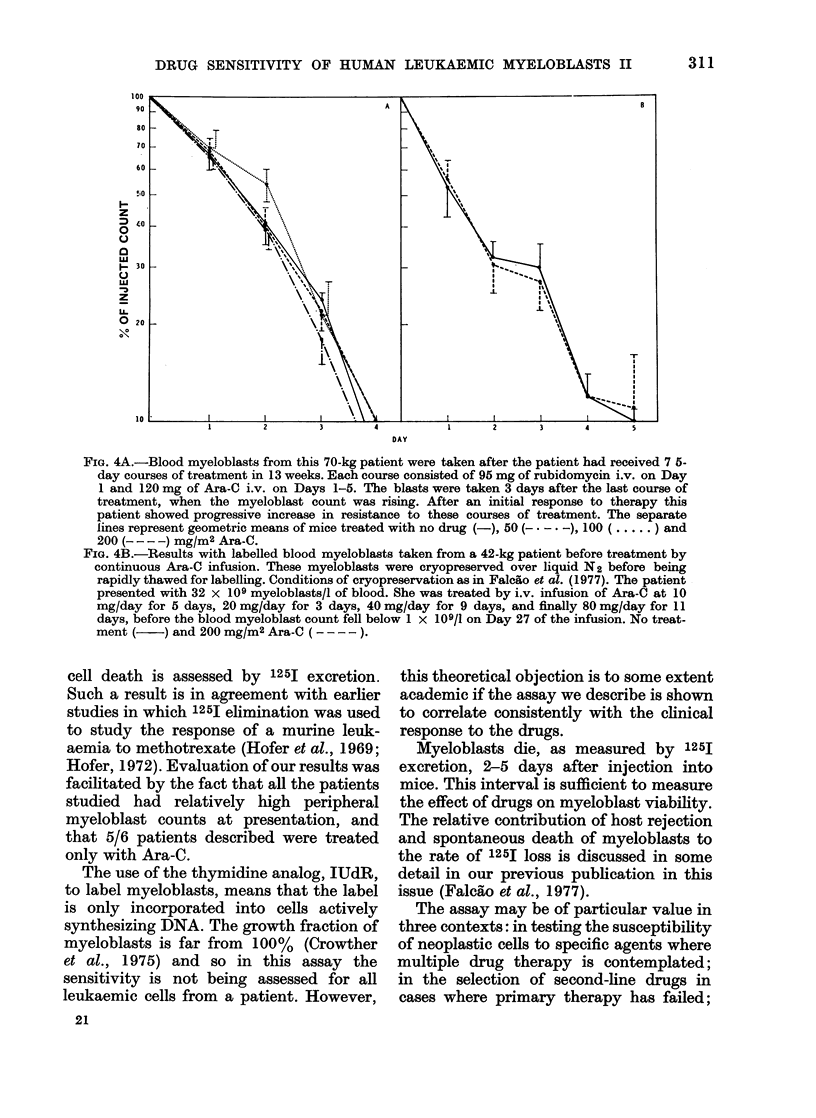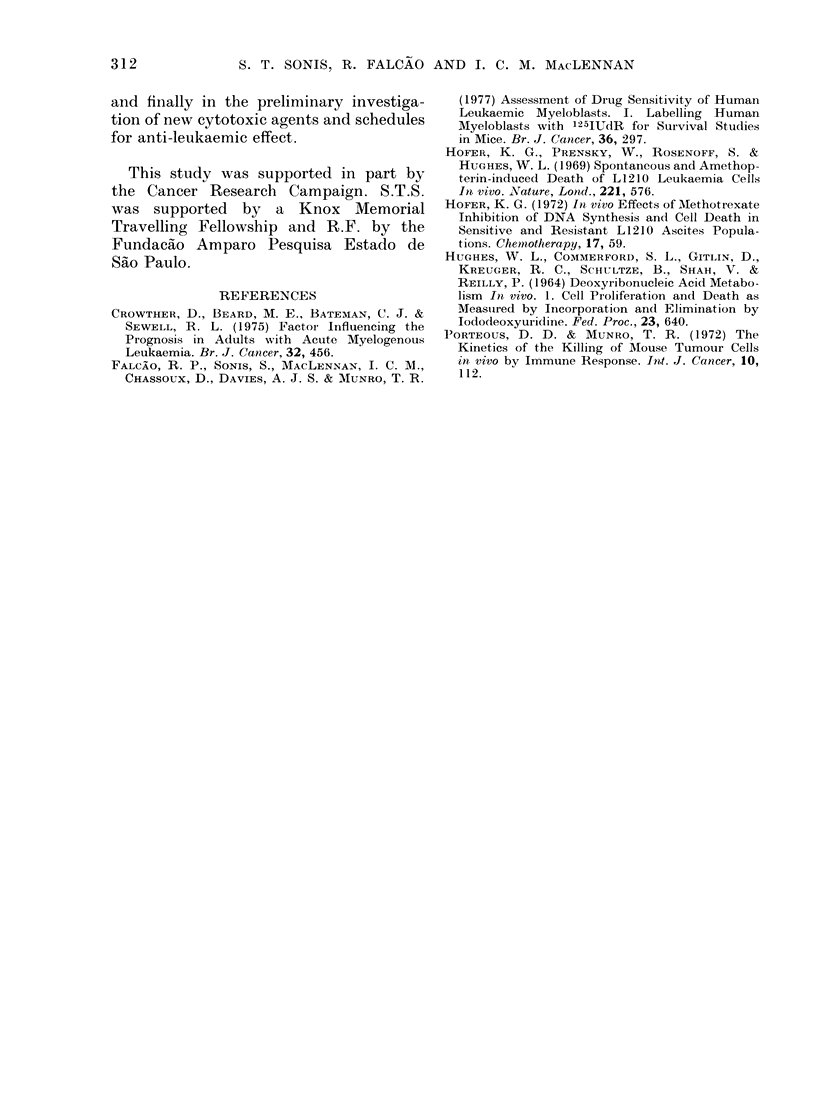# Assessment of drug sensitivity of human leukaemic myeloblasts. II. The toxic effects of cytosine arabinoside on 125IUdR-labelled human leukaemic myeloblasts in mice.

**DOI:** 10.1038/bjc.1977.194

**Published:** 1977-09

**Authors:** S. T. Sonis, R. Falcão, I. C. MacLennan

## Abstract

Leukaemia cells from the peripheral blood and bone marrow of patients with acute myeloblastic leukaemia were labelled in vitro with [125I]5-iodo-2'-deoxy-uridine (IUdR). The myeloblasts were then injected into groups of mice and the survival of these cells estimated by measuring isotope loss, using whole-body counting. The isotope excretion from mice treated with various doses of cytosine arabinoside (Ara-C) and those not treated with drugs were compared. This comparison showed that the sensitivity of myeloblasts to the drug varies from patient to patient, and in one case was different for myeloblasts from bone marrow and from blood from the same patient. We compare the clinical responses of myeloblasts to Ara-C in 6 patients, who had high peripheral blood myeloblast counts, with the sensitivities of their myeloblasts to Ara-C in mice. This comparison indicates that the assay might be a useful way of predicting the response of leukaemic cells in patients to cytotoxic agents.


*Br. J. Cancer* (1977) **36**, 307

### ASSESSMENT OF DRUG SENSITIVITY OF HUMAN LEUKAEMIC MYELOBLASTS

### II. THE TOXIC EFFECTS OF CYTOSINE ARABINOSIDE ON $^{125}$IUdR-LABELLED HUMAN LEUKAEMIC MYELOBLASTS IN MICE

S. T. SONIS*, R. FALCÃO† AND I. C. M. MACLENNAN‡

*From the *Department of Surgery, Peter Bent Brigham Hospital, Boston, Massachusetts, U.S.A., the †Facultade Medicina, Ribeirão Preto, Brazil, and the ‡Nuffield Department of Clinical Medicine, The Radcliffe Infirmary, Oxford OX2 6HE*

Received 12 July 1976 Accepted 25 April 1977

**Summary.—Leukaemia cells from the peripheral blood and bone marrow of patients with acute myeloblastic leukaemia were labelled *in vitro* with [$^{125}$I] 5-iodo-2′-deoxyuridine (IUdR). The myeloblasts were then injected into groups of mice and the survival of these cells estimated by measuring isotope loss, using whole-body counting. The isotope excretion from mice treated with various doses of cytosine arabinoside (Ara-C) and those not treated with drugs were compared. This comparison showed that the sensitivity of myeloblasts to the drug varies from patient to patient, and in one case was different for myeloblasts from bone marrow and from blood from the same patient. We compare the clinical responses of myeloblasts to Ara-C in 6 patients, who had high peripheral blood myeloblast counts, with the sensitivities of their myeloblasts to Ara-C in mice. This comparison indicates that the assay might be a useful way of predicting the response of leukaemic cells in patients to cytotoxic agents.**

MEASUREMENT of $^{125}$I release from [$^{125}$I]5-iodo-2′-deoxyuridine (IUdR)-labelled cells has been used to assess cell survival *in vivo* in a number of animal models (Hughes *et al.*, 1964; Porteous and Munro, 1972). In the previous paper in this series (Falcão *et al.*, 1977) we described a technique for labelling both fresh and cryopreserved human leukaemic myeloblasts with IUdR, and we investigated the fate of the labelled cells after they had been injected into mice. The present study was undertaken to establish, in principle, whether this technique can be used to assess the susceptibility *in vivo* of human leukaemic myeloblasts to chemotherapy.

### PATIENTS

The patients studied had definite acute myelobastic leukaemia. Brief clinical details are given in the legends to figures. We are extremely grateful to Drs C. Bunch, S. Callender, C. Potter and Professor D. Weatherall for allowing us to study their patients. Five of the patients were treated by low-dose cytosine arabinoside (Ara-C) infusion as part of a pilot study being carried out at Oxford by these workers. The rational basis for this treatment and the reasons for dose selection will be described elsewhere.

### MATERIALS AND METHODS

*Myeloblasts.*—Myeloblasts were collected from the peripheral blood or bone marrow. Blood was anticoagulated with either acid citrate, dextrose or heparin. Bone-marrow aspirates were flushed into Medium RPMI 1640 containing heat-inactivated 10% foetal bovine serum. Red blood cells and neutrophils were removed from bone-marrow preparations by centrifugation through a Ficoll–Triosil gradient of sp. gr. 1·080. Interface cells were then washed in RPMI before being

Correspondence to: I. C. M. MacLennan, Nuffield Department of Clinical Medicine, The Radcliffe Infirmary, Oxford OX2 6HE, England.

labelled. Myeloblasts from blood were separated from red cells by 1 *g* sedimentation followed by one wash to remove platelets. Preparations for labelling contained more than 90% myeloblasts by morphological counting of Giemsa-stained films.

*Labelling of myeloblasts.*—A detailed description of the way suitable conditions for myeloblast labelling was established is given in the preceding paper (Falcão *et al.*, 1977). RPMI 1640 containing 200 u/ml of penicillin, 100 $\mu$g/ml of streptomycin, fresh glutamine, 10% pooled human AB serum (heated previously at 56 °C for 30 min) and 0·006 $\mu$Ci/ml of $^{125}$IUdR (Amersham, sp. act. 25–35 Ci/mmol) was used for labelling. Myeloblasts were added to flat-bottomed glass culture flasks at $0{\cdot}7 \times 10^6$ cells/ml. The culture medium was 1–1·5 cm deep in the flasks during labelling, and the flasks were gassed with 5% $CO_2$:95% air. Cultures were incubated for 18–20 h at 37 °C. The cells were then centrifuged and washed twice in Medium RPMI without serum supplement.

*Animals.*—CBA/He T6T6 or C3H mice 8–10 weeks old were used in this study. Groups were matched for weight, and animals were of the same strain and sex in each experiment.

*Labelling BP8 cells.*—BP8 is a C3H-derived fibrosarcoma which is maintained by serial passage as an ascites tumour in C3H mice. BP8 cells were removed from the peritoneal cavity, washed twice in RPMI 1640, counted and suspended at $2 \times 10^5$ cells/ml in RPMI with $^{125}$IUdR at 0·03 $\mu$Ci/ml. The mixture was incubated for 2 h at 37 °C in an atmosphere of 5% $CO_2$:95% air. The cells were then washed twice to remove free label. The cells were then incubated for a further 30 min at 37 °C, rewashed and suspended at $10^7$ cells/ml. Where killed BP8 cells were to be injected, they were freeze–thawed 4 times. Both killed and viable labelled BP8 were injected s.c. at $5 \times 10^6$/mouse.

In vivo *assay procedure.*—Labelled myeloblasts were injected s.c. between the scapulae. Each mouse was given $1\text{–}5 \times 10^7$ labelled myeloblasts, the number of cells depending on the amount of label incorporated by them. Whole-body $^{125}$I was estimated each day at about the same time, by counting the mice in a large NaI well counter. Mice received 0·1% KI in their drinking water for 2 days before myeloblast injection, and throughout the duration of each experiment, to block the uptake of released $^{125}$I by the thyroid. A suspension of labelled myeloblasts in water was counted at the same time as the mice, to determine isotope decay and compensate for fluctuations in counting efficiency. The activity remaining at any time is expressed as a percent of activity at the time of injection of myeloblasts and is calculated:

percent radioactivity remaining at Day $t$ =

$$\frac{\text{Counts Day } t \times \dfrac{(\text{Counts standard Day 0})}{(\text{Counts standard Day } t)}}{\text{Counts Day 0}} \times 100$$

All counts had background subtracted.

Results are expressed as the geometric mean ± $\log_{10}$ s.d. of the per cent radioactivity remaining.

FIG. 1.—The effect of Ara-C on the excretion of $^{125}$I from mice injected with IUdR-labelled and killed BP8 fibrosarcoma cells. The cells were injected s.c. and the drug administered i.v. ●, mice injected with Ara-C 24 h before the cells. ▲, mice injected with Ara-C immediately after the injection of cells.

% inhibition of release =

$$100 \times \frac{\begin{array}{c}\% \ ^{125}\text{I retained in} \\ \text{mice receiving drug} \\ \text{and killed cells}\end{array} - \begin{array}{c}\% \ ^{125}\text{I retained in} \\ \text{mice receiving} \\ \text{killed cells only}\end{array}}{\begin{array}{c}\% \ ^{125}\text{I retained} \\ \text{in mice receiving} \\ \text{live cells}\end{array} - \begin{array}{c}\% \ ^{125}\text{I retained} \\ \text{in mice receiving} \\ \text{killed cells only}\end{array}}$$

Groups of 3 mice were used. The % inhibition of $^{125}$I release is calculated from the geometric mean of the % $^{125}$I retained by the groups 24 h after the injection of BP8.

### RESULTS

*The effect of cytosine arabinoside on the clearance of $^{125}I$ by mice injected with killed IUdR-labelled cells*

This experiment was carried out using $^{125}$IUdR-labelled BP8 fibrosarcoma cells which were killed by repeated freezing and thawing before being injected s.c. into mice. The results are shown in Fig. 1. Ara-C was given i.v. at varying doses, either immediately after or 24 h before, the injection of cells.

When Ara-C was given immediately after BP8 cells, there was no slowing in the rate of $^{125}$I loss from mice. However, 100 or 200 mg Ara-C/m$^2$, when given 24 h before the cells, caused a slowing in the rate of $^{125}$I elimination. By 48 h after cell injection, $^{125}$I retention was not significantly different between treated and untreated mice. Similar results were obtained when this experiment was repeated.

*The effect of Ara-C on the excretion of $^{125}I$ by mice injected with IUdR-labelled human leukaemic myeloblasts*

The results of these experiments are given in Figs 2, 3 and 4.

In Fig. 2A and B, experiments are shown where there was significant increase in the rate of $^{125}$I loss from Ara-C-treated mice. The clinical details given in the legends to figures indicate that these patients showed a rapid response to Ara-C infusion. However, the onset of acute marrow necrosis in the patients whose myeloblasts are represented in Fig. 2B complicates evaluation of the drug sensitivity of his myeloblasts.

Fig. 3A and B show myeloblasts which

FIGS. 2, 3 and 4.—$^{125}$I excretion from mice injected s.c. with $^{125}$IUdR-labelled human leukaemic myeloblasts. The effect of an i.v. injection of Ara-C immediately after the injection of cells on $^{125}$I excretion is assessed. Geometric means and logarithmic s.d. are shown for groups of 3–5 mice.

FIG. 2A.—This 37·5kg patient's blood myeloblasts were taken immediately before treatment. At this stage she had 56 × 10$^9$ leukaemic myeloblasts per litre of blood. She was given 20 mg of Ara-C per day by continuous i.v. infusion and by the 12th day the blood myeloblast count had fallen to 1 × 10$^9$/l. ——, no drug; – – – –, 200 mg Ara-C/m$^2$. – · – · – 400 mg Ara-C/m$^2$.

FIG. 2B.—This 72·5kg patient presented with a blood myeloblast count of 63 × 10$^9$/l. Bone marrow aspiration at that time only yielded dark brown necrotic tissue at three sites. He was treated with continuous i.v. infusion of Ara-C 20 mg/day for 2 days and 80 mg/day for 5 days, before his blood myeloblast count fell to 1 × 10$^9$/l. Blood myeloblasts were studied immediately before treatment. ———— = no drug. – · – · – = 50 mg/m$^2$ Ara-C. ...... = 100 mg/m$^2$ Ara-C.

FIG. 3A.—Blood myeloblasts were taken from this 65-kg patient immediately before treatment by continuous i.v. infusion of Ara-C. After 28 days' infusion at 12 mg Ara-C/day, his blood myeloblast count fell from $35 \times 10^9$ to $1 \times 10^9$/l. ——— = no drug. — · — · — = 50 mg/m² Ara-C. . . . . . = 100 mg/m² Ara-C.

FIG. 3B and C.—Blood myeloblasts (Fig. 3B) and bone marrow myeloblasts (Fig. 3C) taken from this 92-kg patient immediately before treatment. This patient presented with $22 \times 10^9$ myeloblasts/l of blood and was treated by continuous i.v. infusion of Ara-C at 20 mg/day for 2 days, 22 mg/day for 24 days and then 44 mg/m² for 13 days before his blood myeloblast count fell to $1 \times 10^9$/l. ——— = no drug. — · — · — = 50 mg/m²/day. . . . . . = 100 mg/m²/day.

have a high spontaneous rate of IUdR release. In each case, about 90% of injected radioactivity is lost within 48 h. This high rate of isotope release is probably due to rapid death of the labelled cells. It is gratifying to see that despite this, a significant cytotoxic effect of Ara-C is demonstrable in mice, against the myeloblasts of the patient depicted in Fig. 3A. Fig. 3B and C indicate that different characteristics may be seen in myeloblasts from a single individual taken from different sites. The marrow myeloblasts from this patient represented in Fig. 3C have a low rate of $^{125}$I loss, and these cells appear sensitive to damage by Ara-C. On the other hand the same patient's peripheral blood myeloblasts (Fig. 3B) show more rapid spontaneous release of isotope, and this is not increased further by the administration of Ara-C. We have compared marrow and blood myeloblasts in 2 other patients. In these patients the drug sensitivity and spontaneou srate of isotope release did not differ between cells from the two sites. In all 3 patients the number of counts taken up by marrow cells was greater than that by blood cells.

Fig. 4 shows the rate of excretion of $^{125}$I from mice injected with IUdR-labelled myeloblasts from 2 further patients. The spontaneous rate of isotope release is not particularly high from the myeloblasts from either patient, but in neither case did Ara-C increase isotope release. The clinical history of the response to Ara-C in these 2 patients, which is given in the legend to Fig. 3, shows that neither patient was markedly sensitive to this drug.

### DISCUSSION

Our results, although obtained in a small group of patients, indicate that it is feasible to measure the susceptibility of leukaemic myeloblasts to lysis by chemotherapeutic agents, using an assay in which

FIG. 4A.—Blood myeloblasts from this 70-kg patient were taken after the patient had received 7 5-day courses of treatment in 13 weeks. Each course consisted of 95 mg of rubidomycin i.v. on Day 1 and 120 mg of Ara-C i.v. on Days 1–5. The blasts were taken 3 days after the last course of treatment, when the myeloblast count was rising. After an initial response to therapy this patient showed progressive increase in resistance to these courses of treatment. The separate lines represent geometric means of mice treated with no drug (—), 50 (– · – · –), 100 ( . . . . . ) and 200 (– – – –) mg/m² Ara-C.

FIG. 4B.—Results with labelled blood myeloblasts taken from a 42-kg patient before treatment by continuous Ara-C infusion. These myeloblasts were cryopreserved over liquid $N_2$ before being rapidly thawed for labelling. Conditions of cryopreservation as in Falcão *et al.* (1977). The patient presented with $32 \times 10^9$ myeloblasts/l of blood. She was treated by i.v. infusion of Ara-C at 10 mg/day for 5 days, 20 mg/day for 3 days, 40 mg/day for 9 days, and finally 80 mg/day for 11 days, before the blood myeloblast count fell below $1 \times 10^9$/l on Day 27 of the infusion. No treatment (——) and 200 mg/m² Ara-C ( – – – – ).

cell death is assessed by $^{125}$I excretion. Such a result is in agreement with earlier studies in which $^{125}$I elimination was used to study the response of a murine leukaemia to methotrexate (Hofer *et al.*, 1969; Hofer, 1972). Evaluation of our results was facilitated by the fact that all the patients studied had relatively high peripheral myeloblast counts at presentation, and that 5/6 patients described were treated only with Ara-C.

The use of the thymidine analog, IUdR, to label myeloblasts, means that the label is only incorporated into cells actively synthesizing DNA. The growth fraction of myeloblasts is far from 100% (Crowther *et al.*, 1975) and so in this assay the sensitivity is not being assessed for all leukaemic cells from a patient. However, this theoretical objection is to some extent academic if the assay we describe is shown to correlate consistently with the clinical response to the drugs.

Myeloblasts die, as measured by $^{125}$I excretion, 2–5 days after injection into mice. This interval is sufficient to measure the effect of drugs on myeloblast viability. The relative contribution of host rejection and spontaneous death of myeloblasts to the rate of $^{125}$I loss is discussed in some detail in our previous publication in this issue (Falcão *et al.*, 1977).

The assay may be of particular value in three contexts: in testing the susceptibility of neoplastic cells to specific agents where multiple drug therapy is contemplated; in the selection of second-line drugs in cases where primary therapy has failed; and finally in the preliminary investigation of new cytotoxic agents and schedules for anti-leukaemic effect.

This study was supported in part by the Cancer Research Campaign. S.T.S. was supported by a Knox Memorial Travelling Fellowship and R.F. by the Fundacão Amparo Pesquisa Estado de São Paulo.

### REFERENCES

CROWTHER, D., BEARD, M. E., BATEMAN, C. J. & SEWELL, R. L. (1975) Factor Influencing the Prognosis in Adults with Acute Myelogenous Leukaemia. *Br. J. Cancer*, **32**, 456.

FALCÃO, R. P., SONIS, S., MACLENNAN, I. C. M., CHASSOUX, D., DAVIES, A. J. S. & MUNRO, T. R. (1977) Assessment of Drug Sensitivity of Human Leukaemic Myeloblasts. I. Labelling Human Myeloblasts with $^{125}$IUdR for Survival Studies in Mice. *Br. J. Cancer*, **36**, 297.

HOFER, K. G., PRENSKY, W., ROSENOFF, S. & HUGHES, W. L. (1969) Spontaneous and Amethopterin-induced Death of L1210 Leukaemia Cells *In vivo*. *Nature, Lond.*, **221**, 576.

HOFER, K. G. (1972) *In vivo* Effects of Methotrexate Inhibition of DNA Synthesis and Cell Death in Sensitive and Resistant L1210 Ascites Populations. *Chemotherapy*, **17**, 59.

HUGHES, W. L., COMMERFORD, S. L., GITLIN, D., KREUGER, R. C., SCHULTZE, B., SHAH, V. & REILLY, P. (1964) Deoxyribonucleic Acid Metabolism *In vivo*. 1. Cell Proliferation and Death as Measured by Incorporation and Elimination by Iododeoxyuridine. *Fed. Proc.*, **23**, 640.

PORTEOUS, D. D. & MUNRO, T. R. (1972) The Kinetics of the Killing of Mouse Tumour Cells *in vivo* by Immune Response. *Int. J. Cancer*, **10**, 112.